# New insights into fibrotic signaling in renal cell carcinoma

**DOI:** 10.3389/fcell.2023.1056964

**Published:** 2023-02-21

**Authors:** Jiao-Yi Chen, Wai-Han Yiu, Patrick Ming-Kuen Tang, Sydney Chi-Wai Tang

**Affiliations:** ^1^ Division of Nephrology, Department of Medicine, The University of Hong Kong, Hong Kong, Hong Kong SAR, China; ^2^ Department of Anatomical and Cellular Pathology, State Key Laboratory of Translational Oncology, The Chinese University of Hong Kong, Hong Kong, China

**Keywords:** renal fibrosis, renal cell carcinoma, cancer-associated fibroblast, mTOR, TGF-β

## Abstract

Fibrotic signaling plays a pivotal role in the development and progression of solid cancers including renal cell carcinoma (RCC). Intratumoral fibrosis (ITF) and pseudo-capsule (PC) fibrosis are significantly correlated to the disease progression of renal cell carcinoma. Targeting classic fibrotic signaling processes such as TGF-β signaling and epithelial-to-mesenchymal transition (EMT) shows promising antitumor effects both preclinically and clinically. Therefore, a better understanding of the pathogenic mechanisms of fibrotic signaling in renal cell carcinoma at molecular resolution can facilitate the development of precision therapies against solid cancers. In this review, we systematically summarized the latest updates on fibrotic signaling, from clinical correlation and molecular mechanisms to its therapeutic strategies for renal cell carcinoma. Importantly, we examined the reported fibrotic signaling on the human renal cell carcinoma dataset at the transcriptome level with single-cell resolution to assess its translational potential in the clinic.

## 1 Introduction

Renal cell carcinoma accounts for approximately 3% of all adult malignant diseases and over 90% of kidney cancer (Ferlay et al., 2015). In 2020, the new cases of kidney cancer have reached 431,288 worldwide, with 179,368 related deaths reported accordingly (Siegel et al., 2021). Although the 5-year survival is 60% overall for kidney cancer, it drops to 10% in patients with metastasis (Sengupta et al., 2005; Bianchi et al., 2012; Howlader et al., 2019). Currently, over 10 histological and molecular subtypes of RCC have been identified (Lopez-Beltran et al., 2006; Moch et al., 2016), of which clear cell renal cell carcinoma (ccRCC) is the most common RCC subtype (75% in all RCCs) (Creighton et al., 2013), followed by papillary RCC (pRCC, about 15% in all RCCs) (Linehan et al., 2016) and chromophobe RCC (chRCC, about 5% in total RCCs) (Davis et al., 2014). The discovery of von Hippel–Lindau (VHL) and other epigenetic regulatory gene mutations further advanced the knowledge of RCC development (Creighton et al., 2013; Alaghehbandan et al., 2019; Lin et al., 2021). However, how immune escape and distant metastasis initiate in RCC remains obscure.

Cancer-associated fibrosis was found to play a critical role in tumorigenesis, immune evasion, metastasis, and drug resistance in various solid tumors (Coffman et al., 2016; Kalluri, 2016; Piersma et al., 2020). Epidemiological findings strongly indicate a prognostic relevance between tissue fibrosis and epithelial cancers, such as hepatic, gastroesophageal, lung, and renal cancers (Neglia et al., 1995; Leek et al., 1996; Maisonneuve et al., 2013; Joung et al., 2018; Ballester et al., 2019). Moreover, fibrosis in the tumor immune microenvironment (TME), characterized by ITF (Chandler et al., 2019; Liu et al., 2019), activation of cancer-associated fibroblasts (CAFs) (Kalluri, 2016), and extracellular matrix (ECM) deposition (Chandler et al., 2019), supports tumor growth by producing growth factors, stimulating angiogenesis (Ziani et al., 2018; Inoue et al., 2019). Fibrotic signaling can also mediate immunosuppression by facilitating Treg cell and myeloid-derived suppressor cell (MDSC) differentiation and recruitment (Costa et al., 2018; Givel et al., 2018; Lin et al., 2022), metabolic reprogramming (Hamanaka and Mutlu, 2021), and long non-coding RNA (lncRNA) regulation (Shi et al., 2021). Therefore, fibrotic signaling has become a promising therapeutic target for cancer.

In this review, we summarized the pathogenic roles and underlying mechanisms of various RCC-associated fibrosis in the development and progression of RCC. In addition, we validated the reported findings in RCC patients datasets by using gene set enrichment analysis (GSEA) and single-cell RNA sequencing (scRNA-seq) data mining. Furthermore, the translational potentials of new therapeutic strategies targeting fibrotic signaling in RCC were also discussed.

## 2 Prognostic relevance between fibrosis and RCC

Fibrosis is a common feature frequently observed in RCC (Cheville et al., 2003), characterized by ITF, CAFs, ECM deposition, peritumoral PC fibrosis, and EMT. CAFs play an essential and versatile role in all stages of RCC. They are a group of robustly proliferative and metabolically activated fibroblasts (Guido et al., 2012; Kalluri, 2016), characterized by high expression of α-smooth muscle actin (α-SMA), fibroblast activation protein (FAP), S100A4, and platelet-derived growth factor receptors α and β (PDGFα and PDGFβ) (Kalluri, 2016). CAFs can be derived from multiple sources including resident fibroblasts (Kojima et al., 2010), mesenchymal stem cells (MSCs) (Barcellos-de-Souza et al., 2016), and epithelial and endothelial cells (Petersen et al., 2003; Yeon et al., 2018). Mechanistically, CAFs continuously interplay with tumor cells by producing pro-tumor cytokines, activating immunosuppressive leukocytes, and promoting ECM deposition, thus offering a tumor-favorable microenvironment (Kalluri, 2016). A recent cohort study revealed that the CAFs are significantly correlated with shorter disease-free survival (DFS), poorer overall survival (OS), and lymph node metastasis among ccRCC patients (Ambrosetti et al., 2022). Xu et al. further specified CD248 + CAFs as the pivotal CAF phenotype that was remarkably related to poor prognosis and immunosuppressive TME during RCC progression (Xu et al., 2021).

CAFs can directly form fibrotic tumor stroma *via* cross-linked collagen matrix deposition. Such ITF is associated with decreased lymphocyte infiltration, poorer patient survival, and various carcinomas, including breast cancer (Solinas et al., 2017; Li et al., 2019), lung cancer (Ballester et al., 2019), colorectal cancer (Nazemalhosseini-Mojarad et al., 2019), pancreatic cancer (Sinn et al., 2014), and advanced rectal cancer (Ueno et al., 2004). A retrospective cohort study involving 204 RCC patients found that over 80% of ccRCC cases had intratumoral fibrosis (Joung et al., 2018). Although ITF itself does not have a significant association with ccRCC prognosis, it is correlated with other prognostic factors such as Fuhrman nuclear grade, intratumoral necrosis, and lymphovascular invasion (Joung et al., 2018).

CAFs are also the major source of tumor-associated ECM (Bond et al., 2021). A recent proteomics study revealed that the composition of ECM in ccRCC varies significantly from their respective counterparts in the neighboring healthy cortex. RCC-associated ECM is more abundant, denser, and stiffer, with increased deposition of fibronectin (*FN1*), collagen 1 (*COL1A1* and *COL1A2*), and collagen 6 (*COL6A1*, *COL6A2*, and *COL6A3*) (Bond et al., 2021). Also, some of these overexpressed fibrotic ECM proteins, including fibronectin 1 and collagen 1, are correlated with a poorer prognosis among ccRCC and pRCC patients (Steffens et al., 2012; Majo et al., 2020).

Interestingly, in contrast to the previous findings, several studies show that tumor-related fibrosis might limit tumor growth and metastasis at the early stages of cancer (Bruno et al., 2013; Alkasalias et al., 2014). In terms of RCC, such protective fibrosis is referred to as the fibrotic PC (Xi et al., 2018). PC is a common pathologic feature that exists in almost all the early-stage RCCs and is composed of fibrous tissue, compressed normal renal tissue, and scaffolding of vascular tissues in the RCC surrounding area (Huang et al., 1992; Tsili et al., 2012; Minervini et al., 2014; Cheng et al., 2015; Cho et al., 2017). The frequency of PC appearance varies from 33% to 72% in RCC. As the only barrier interposing between RCC and the surrounding normal renal parenchyma, the intact PC indicates a limited tumor-to-immune cell and tumor-to-matrix interaction in the TME and lower aggressiveness of RCCs. Several clinical observations revealed that the tumor invasion of the PC suggested a poor prognosis with a higher risk of local recurrence and metastasis (Yamashita et al., 1996; Cho et al., 2009; Xi et al., 2018). Qin et al. (2020) mentioned that fibrosis in PCs might strengthen the vital barriers to prevent tumor penetration. By quantifying collagen distribution in PCs among RCC patients, they further noticed that fibrosis in PCs is an independent marker of PC integrity. Lower PC fibrosis is significantly associated with shorter progression-free survival.

## 3 Transcriptome profile uncovered enriched fibrotic signaling in RCC

With the emerging RNA sequencing technologies, from bulk RNA sequencing (bulk RNA-seq) to scRNA-seq, transcriptome profiling of RCC has been greatly utilized in biomarker discovery, cancer heterogeneity characterization, and studies regarding distant metastasis and therapy resistance. More importantly, understanding the role of fibrosis in RCC development enlightens further therapeutic target identification by data mining the transcriptomic database of RCC patients. Here, we performed gene set enrichment analysis, as previously described (Chen et al., 2022), of a public human RCC scRNA-seq dataset (Young et al., 2018) and summarized the activated fibrotic signaling pathways identified in different subtypes and stages of human RCC.

Early in 2010, López-Lago et al. (2010) used RNA sequencing to provide evidence for the association of increased metastatic activity with the acquisition of a myofibroblast-like feature in both RCC cell lines and human metastatic RCC biopsies. Later, activation of the pro-fibrotic TGF-β signaling pathway was further identified in the transcriptome profile of MiTF/TFE translocation RCC (Malouf et al., 2014). Another two bulk RNA-seq datasets, comparing mRNA expression between human ccRCC and normal kidney tissue, also revealed pro-fibrosis signatures *ACTA2* (α-SMA), *COL1A1*, *COL23A1*, *VEGFA*, and *TGFB1* as the top differentially expressed genes (DEGs) upregulated in ccRCC (Xiong et al., 2014; Eikrem et al., 2016). Elevated TGF-β signaling was also identified in the transcriptomic profile of 176 ccRCC patients, correlated with poor disease survival and tumor metastasis (Zhao et al., 2006; Sjölund et al., 2011). Sven Wach et al. (2019) reported over 5,000 DEGs with the criteria |log_2_ fold change| ≥1 in collecting duct carcinoma (CDC), in comparison with the normal kidney tissue. Enrichment analysis targeting those DEGs further identified the pro-fibrosis collagen signaling pathway as the top-ranked enriched pathway activated in the CDC group. *KRT17* (keratin 17), a wounded stratified epithelium-induced filament protein, was identified as the top DEG with the highest fold change of expression in CDC [Wach et al. (2019)]. More recently, *KRT17* high-expressing basal-like cells were defined in fibrotic hypersensitivity pneumonitis patients with higher expression levels of *COL1A1*, *FN1*, and *COL6A2* and upregulated activities in ECM organization by scRNA-seq analysis (Wang et al., 2022b). Findings of the scRNA-seq profile, in combination with the CDC bulk RNA profile, suggest a potential pathogenic role of KRT17-mediated fibrosis during RCC development.

**FIGURE 1:**
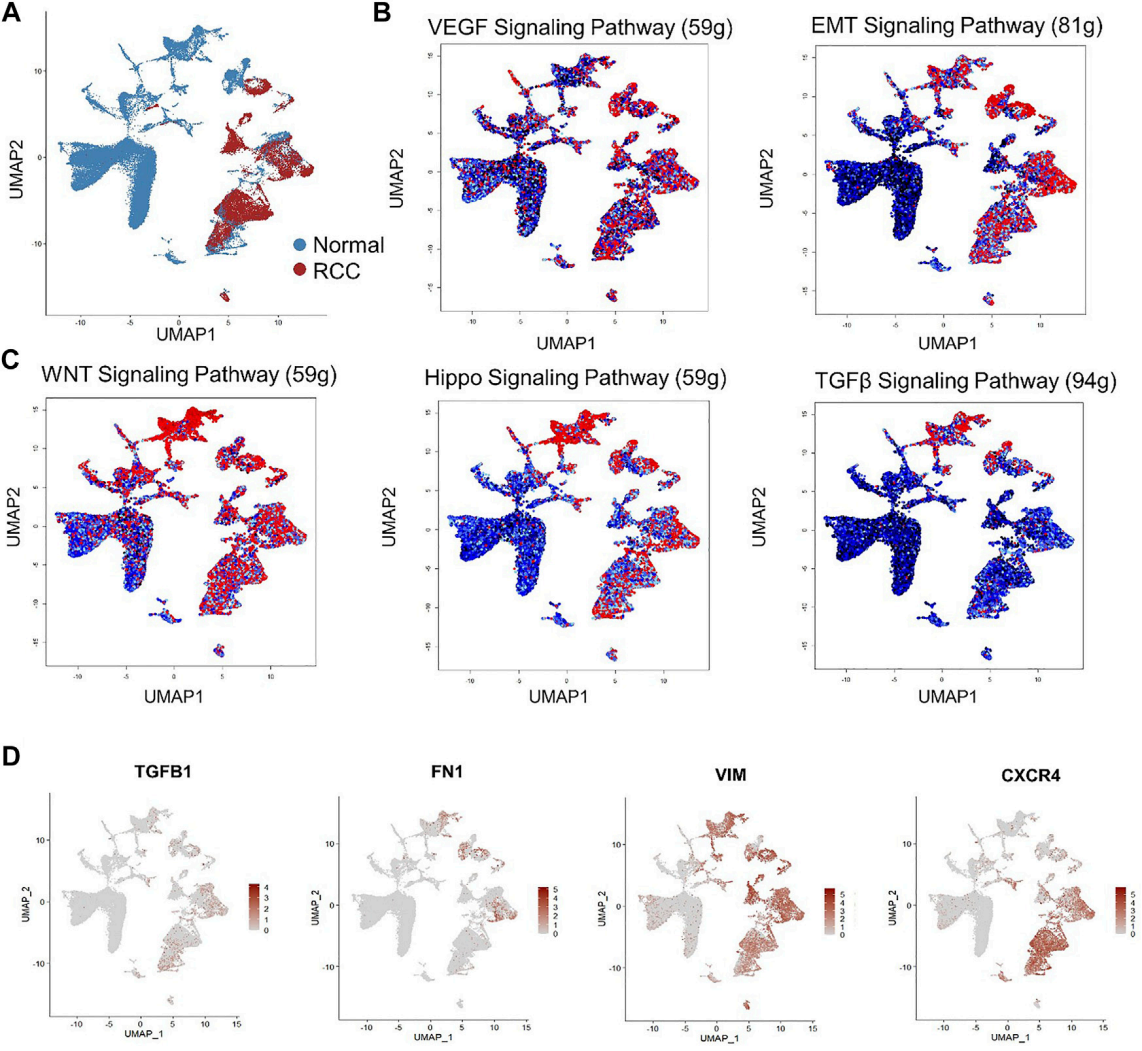
Single-cell RNA sequencing (scRNA-seq) reveals activated fibrotic signaling pathways in human renal cell carcinoma. **(A)** Non-linear dimensionality reduction on Uniform Manifold Approximation and Projection (UMAP) visualization of renal cells from RCC patients and the healthy control group. Each point depicts a single cell, colored according to group designation. Colored UMAP plots of highlighted cells with activated gene set expression in **(B)** VEGF and EMT signaling and **(C)** WNT, Hippo, and TGF-β signaling pathways in renal cells based on AUC scores. Each point represents a single cell. Cells with indicated signaling activation are colored in shades of red and those without signaling activation are colored in black–blue. **(D)** UMAP plots of gene expression gradients identified. Each point depicts a single cell, colored according to normalized expression levels. The average expression scale is shown on the right side of each UMAP plot.

Nevertheless, the aforementioned studies mainly focused on biomarker identification and RCC heterogenetic phenotype characterization without an in-depth study of fibrotic signaling participation in RCC at single-cell resolution. To address this question, we performed GSEA of a published human RCC scRNA-seq dataset (Young et al., 2018) downloaded from the European Genome-phenome Archive (EGA) under study IDs EGAS00001002171, EGAS00001002486, EGAS00001002325, and EGAS00001002553. Subsequently, raw UMI counts of the scRNA-seq dataset were imported into R (version 4.1.2) using the Seurat package (version 4.0.5) for quality control. Next, sequencing data from normal control and RCC patients were normalized, scaled, and mapped *via* non-linear dimensionality reduction on the Uniform Manifold Approximation and Projection (UMAP) plot by using the Normalized, ScaleData, and FindVariableFeatures functions, respectively, in the Seurat package to map the comprehensive cell landscape as shown in Figure 1A. Next, fibrosis-related gene set enrichment was tested using GSEABase (version 1.56.0) and GSVA R packages (version 1.42.0) within a panel of annotated gene databases (Gene Ontology, Reactome, and Kyoto Encyclopedia of Genes and Genomes). The enrichment of gene sets at the single-cell level was visualized using the AUCell (version 1.16.0) R Bioconductor package. The transcriptomes of RCC cells were significantly enriched in pro-fibrotic EMT and VEGF signaling (Figure 1B). Fibrotic signaling pathways including WNT and Hippo signaling pathways were also significantly upregulated in RCC (Figure 1C). Although TGF-β signaling is less enriched in RCC cells, *TGFB1* expression, together with three other key fibrosis regulators, *FN1*, *VIM*, and *CXCR4*, was also elevated under the RCC condition (Figure 1D). Taken together, the above findings provided solid evidence of the activation and participation of fibrotic signaling during RCC progression.

## 4 Mechanism of the interplay between fibrosis and RCC

### 4.1 CAF-centered crosstalk between fibrotic stroma and RCC

**FIGURE 2:**
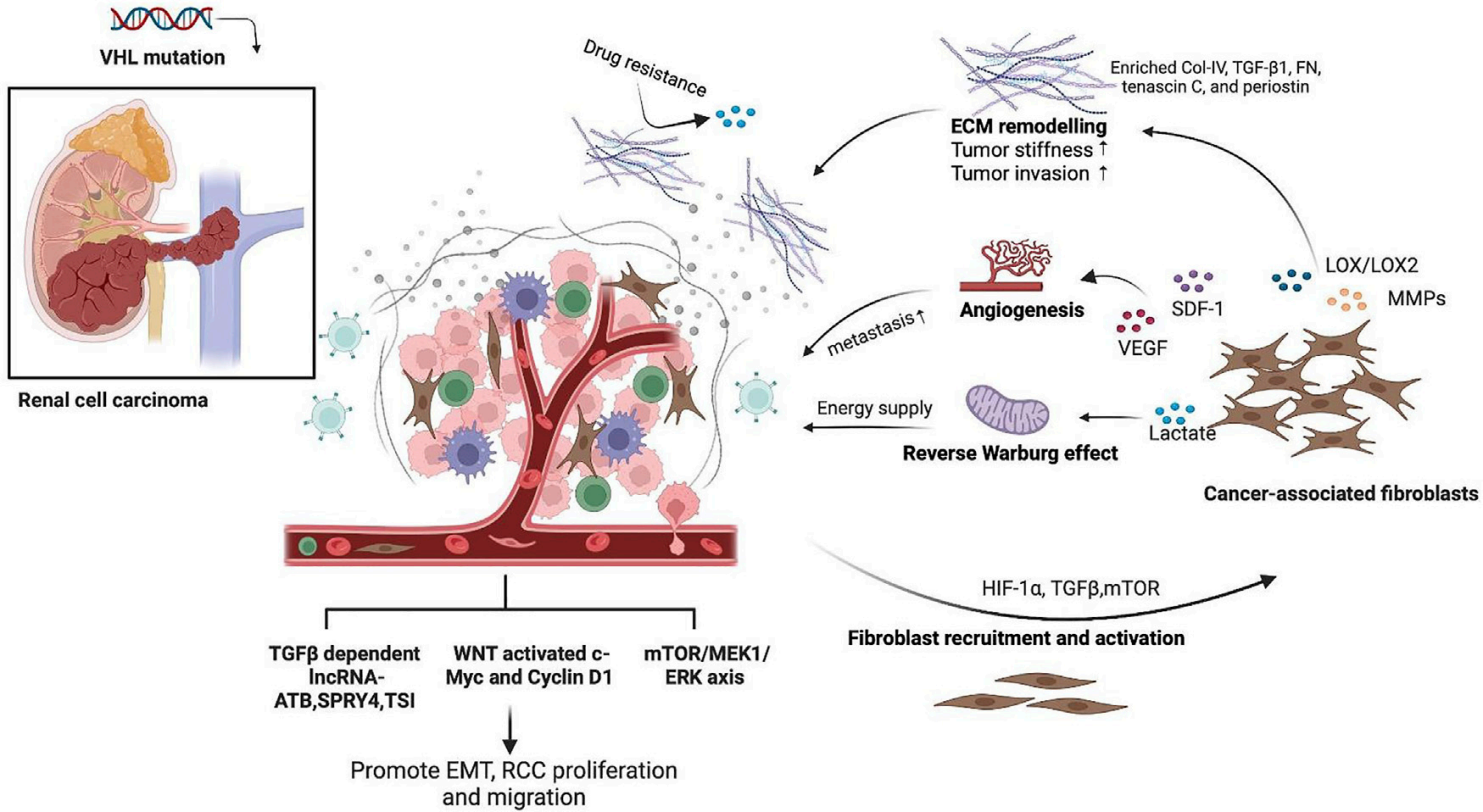
Schematic representation of the role of fibrotic signaling in renal cell carcinoma (RCC). CAFs are the major source of fibrotic stroma in the RCC TME, which could be activated due to HIF-1α accumulation and TGF-β and mTOR signaling regulation from cancer cells. Activated CAFs secreted MMPs and LOX to promote ECM remodeling with enriched collagen IV, TGF-β1, fibronectin, tenascin C, and periostin, thus promoting tumor stiffness, drug resistance, and tumor invasion. CAFs in RCC also produce SDF-1 and VEGF to promote tumor angiogenesis and favor RCC metastasis. CAFs fed cancer cells with increased lactate as a direct energy supply *via* aerobic glycolysis, which is known as the “reverse Warburg effect.” RCC also expressed pro-fibrotic signaling pathways, including TGF-β and its downstream lncRNA, WNT, and mTOR signaling pathways, to mediate the EMT process, tumor cell proliferation, and migration. This figure was created in BioRender.com.

CAFs have long been recognized as a substantial element in the TME that support tumor growth and invasion through diverse mechanisms, including ECM remodeling, cytokine production, immune regulation, and metabolic alteration. In this study, we reviewed and summarized the role of CAF-centered interaction and other critical fibrotic signaling pathways in RCC oncogenesis and metastasis (Figure 2). In RCC, activation of CAFs results from von Hippel–Lindau gene malfunction-induced HIF-1α accumulation (Razorenova et al., 2011; Shen and Kaelin, 2013; Yang et al., 2020). During tumor progression, CAFs continuously produce matrix-crosslinking enzymes, such as LOX family oxidases and matrix metalloproteinases (MMPs), to promote ECM remodeling. This leads to the reorganization of collagen and fibronectin fibers and consequently increases tumor stiffness and contributes to RCC invasion and metastasis (Gilkes et al., 2014). Higher expression levels of LOX family genes, for instance, LOX and LOXL2, indicate poor survival in ccRCC patients (Hase et al., 2014; Lin et al., 2020). Mechanistically, LOX and LOXL2 promote collagen stiffness increment, integrin α5β1 stabilization, and fiber formation while suppressing the protease and proteasome system in ccRCC (Hase et al., 2014). Furthermore, MMPs, including MMP-9 and MMP-2 produced by CAFs, also contribute to tumor invasion and metastasis and have been recognized as potential prognostic biomarkers in ccRCC (Awakura et al., 2006; Chen et al., 2017). In addition, microRNA mir-124-mediated suppression of MMP-9 can attenuate RCC invasiveness *in vitro* (Wang et al., 2018). Regulated by TGF-β1, MMP-13 is more related to bone metastasis of RCC than to primary RCC and healthy kidneys (Kominsky et al., 2008). Moreover, CAFs are also the major producers of the stromal cell-derived factor (SDF-1) (Orimo et al., 2005; Wang et al., 2021). In RCC, CAF-secreted SDF-1 interacts with the chemokine receptor 4 (CXCR4) on renal cancer cells and consequently promotes tumor angiogenesis and organ metabolism (Pan et al., 2006).

CAFs are also capable of transducing the non-muscular myosin II- and PDGFRα-mediated contractility and traction forces to fibronectin *via* integrin α5β1, thus aligning the fibronectin-rich ECM to favor tumor migration (Erdogan et al., 2017; Gopal et al., 2017). Previous scRNA-seq transcriptomic analysis revealed CAF-specific feature expression and identified CAFs as the major sources of ECM in human ccRCC (Young et al., 2018; Bond et al., 2021; Liu et al., 2021). In contrast to the healthy cortex, CAF-contributed ECM production in ccRCC was characterized by high enrichment of collagen VI, fibronectin, tenascin C, TGF-β1, and periostin (Bond et al., 2021). Fibronectin and collagen were the most abundant components in CAF-produced ECM. CAF-induced fibronectin promotes the proliferation and inhibits the apoptosis of precancerous bronchial epithelial and carcinoma cells through the activation of PI3K/AKT signaling in the lung (Han et al., 2006; Han and Roman, 2006). As for RCC patients, higher fibronectin 1 expression showed an increased disease-related mortality rate (Steffens et al., 2012) and a more advanced clinical stage (Dong et al., 2021). Competing endogenous RNA networks further proved the direct correlations between FN1 and C3, FN1 and pro-fibrotic signaling pathways, including WNT, HIF, PI3K/AKT, MAPK, and TGF-β pathways, in ccRCC (Dong et al., 2021). The TGF-β1/Src axis is a well-studied pro-fibrotic signaling pathway that promotes renal fibrosis and tumor progression by promoting macrophage-to-myofibroblast transition (MMT) (Meng et al., 2016; Tang et al., 2018). An *in vitro* study on human RCC cells showed that silencing fibronectin *in vitro* attenuated cell proliferation and migration by suppressing TGF-β1/Src signaling (Ou et al., 2019). Collagen I, another essential CAF-produced ECM component, also facilitates EMT by upregulating MMP-2 and transcription factors ZEB2 and SNAIL in multiple RCC cell lines (Majo et al., 2020), which consequently enhances the proliferation, adhesion, and migration of RCC.

CAFs also influence tumor behavior by reprograming cancer cell metabolism (Martinez-Outschoorn et al., 2014). Aerobic glycolysis, also known as the Warburg effect (Warburg, 1925), has long been recognized as the hallmark metabolic pathway in various solid tumors, including RCC (Singer et al., 2011). During cancer development, anabolic cancer cells rapidly interact with the neighboring CAFs through metabolite exchange and oxidative stress induction and thus consequently promote aerobic glycolysis in CAFs. Such a “dual chamber” process is proposed as a CAF-dependent “reverse Warburg effect” (Pavlides et al., 2009; Benny et al., 2020). To be specific, aerobic glycolysis occurring within CAFs under Warburg metabolism results in lactate generation and deposition in RCC TME. Increased lactate not only acts as a major metabolic fuel for cancer cells directly (Guo et al., 2019), but also contributes to RCC progression and metastasis through indirect mechanisms. Accelerated lactate leads to acidification in TME, which consequently suppresses T-cell cytotoxicity against RCC (Sun et al., 2022). In addition, lactate also accelerates angiogenesis (Trabold et al., 2003), promotes EMT (Miranda-Gonçalves et al., 2020), and weakens cancer sensitivity to programmed cell death (Daneshmandi et al., 2019), thereby enhancing tumor aggressiveness.

CAFs in RCC expressed common fibrotic signaling pathways, including TGF-β, mTOR, MAPK, and WNT/β-catenin signaling pathways, which are similar to those found in most solid tumors (Wu et al., 2021a). Meanwhile, it is increasingly recognized that CAFs and their fibrotic signaling exhibit phenotypic and functional heterogeneity in different tissues/organs and origins. For example, CAFs in lung cancer express distinctively high levels of elastin and collagen (Hao et al., 2019a). Diverse CAF subpopulations with a distinguished transcriptome profile have been recognized in breast cancer (Bartoschek et al., 2018; Costa et al., 2018). Knowledge of CAF heterogeneity will help develop a precision cancer treatment. However, our understanding of RCC-specific CAFs is still very limited. A recent study combining scRNA-seq and cell line sequencing profile has identified the transcriptome signatures of RCC-specific CAFs, including COL1A1, COL1A2, COL5A1, COL16A1, elastin microfibril interfacer 1 (EMILIN1), lysyl oxidase-like 1 (LOXL1), and lumican (LUM) (Liu et al., 2020). Functional enrichment analysis indicates that RCC-specific CAF signatures are significantly associated with extracellular matrix function, collagen synthesis, cell surface interaction, and cell adhesion. Further studies are required to validate the phenotype and functional heterogeneity of CAFs among RCC patients.

### 4.2 TGF-β-centered regulation of fibrosis in RCC progression

TGF-β is the master regulator of renal fibrosis and immune escape in renal cancer (Meng et al., 2015; Chung et al., 2021). Early in 1998, Wunderlich et al. (1998) observed that the latent TGF-β1 was commonly and significantly elevated among RCC patients and even higher among those with pyelonephritis, indicating the oncogenetic role of TGF-β in RCC. With the emerging in-depth studies targeting the pathological role of TGF-β in cancer development, researchers have revealed the dual roles of TGF-β and its downstream cascades during cancer development (Massagué, 2008; Kubiczkova et al., 2012; Chan et al., 2022; Tang et al., 2022). Under normal conditions or at precancerous stages, TGF-β mainly exhibits antitumor activities by maintaining tissue homeostasis and inducing cell cycle arrest to regulate epithelial cell differentiation and apoptosis (Song, 2007; Xu et al., 2018). Nevertheless, cancer cells can always find ways to bypass TGF-β-mediated inhibition, in turn taking advantage of TGF-β signaling or directly producing TGF-β to benefit themselves (Biswas et al., 2014; Yang et al., 2016a; Tang et al., 2017; Rasti et al., 2021). In TME, TGF-β is the key mediator of EMT during tumor progression (Hao et al., 2019b; Xue et al., 2020; Chung et al., 2021). Stimulation with recombinant TGF-β1 significantly promotes EMT in renal carcinoma cells *in vitro* (Boström et al., 2012; Tretbar et al., 2019). Inhibition of TGF-β-induced EMT resulted in suppressed metastasis capacity of RCC (Boström et al., 2013; Wang et al., 2020). TGF-β also promotes EMT by regulating long non-coding RNA (lncRNA) expression in RCC TME. TGF-β1-dependent activation of lncRNA-ATB enhances EMT and tumor cell invasion in hepatocellular carcinoma (Yuan et al., 2014). In RCC, lncRNA-ATB was found to be positively correlated with metastasis and poorer prognosis (Xiong et al., 2016; Qi et al., 2017). Mechanistically, lncRNA-ATB downregulates the tumor suppressor p53 through the regulation of the DNA methyltransferase enzyme DNMT1 and thus strengthens the proliferation and migration of RCC cells (Song et al., 2019). Meanwhile, silencing lncRNA-ATB represses EMT in RCC by suppressing the expression of mesenchymal signatures such as N-cadherin and vimentin (Xiong et al., 2016). Another TGF-β1-induced lncRNA SPRY4-IT1 also demonstrates similar pro-EMT functions both in human RCC and RCC cells *in vitro* (Zhang et al., 2014).

### 4.3 ITF shapes the immune landscape of RCC

Immune cells in TME are considered critical and versatile players in tumor development. Fibrotic tumoral stroma is a significant source of immunosuppressive activity in the TME (Yamauchi et al., 2018; Baker et al., 2021). By producing CXCL12, activated CAFs directly inhibit cytotoxic T-cell recruitment and upregulate immunosuppressive Treg cell infiltration *via* binding to the CXCR4 receptor on cytotoxic T cells and Treg cells in pancreatic cancer (Feig et al., 2013). Yang et al. (2016b) found that FAP + CAFs are the major producers of CCL2 through the uPAR-induced FAP/STAT3/CCL2 axis in the murine liver cancer model. CAF-derived CCL2 in turn induces the recruitment of MDSCs by interacting with CCR2 on circulating MDSCs, thus favoring tumor evasion from immune surveillance. Other molecules produced by CAFs such as MMPs, latent TGF-β, IL-10, and VEGFA were also found to reshape the immune system toward a pro-tumoral pattern (Baker et al., 2021). In addition, CAFs contribute to ECM remodeling by producing MMPs, fibronectin, and collagen, thus increasing ECM stiffness around tumors (Acerbi et al., 2015; Altorki et al., 2019). Consequently, the thickened ECM prevents T-cell infiltration and antitumor drug delivery to cancer cells. Till now, there are still very limited evidence regarding the fibrotic stroma and its interaction with immune activity in TME of kidney cancer. A recent cohort study of 45 ccRCC patients uncovered a significant association between intratumoral fibrosis and cytotoxic T-lymphocyte-associated protein 4 (CTLA4) expression (Han et al., 2022), indicating the potential regulatory role of fibrosis in the tumor immune microenvironment of RCC. However, the underlying mechanism of fibrosis interaction with TME of RCC needs further investigation.

### 4.4 The role of other fibrosis signaling in RCC tumorigenesis

Except for TGF-β/Smad signaling, other pro-fibrotic pathways such as WNT, mTOR, and NOTCH signaling pathways also contribute to tumorigenesis in renal cancer (Table 1). The WNT/β-catenin pathway is one of the most well-studied signaling pathways in renal fibrosis and is activated in human fibrotic chronic kidney disease and the unilateral ureteral obstruction (UUO) mouse model (Tan et al., 2014; Huffstater et al., 2020). In response to injury, renal cells from the tubular epithelium or interstitium ubiquitously produce WNT ligands, such as WNT1, WNT7A, and WNT10A (He et al., 2011; Kuma et al., 2014; Huffstater et al., 2020). The activation of WNT signaling upregulates β-catenin-mediated downstream gene expression, including fibronectin, Snail 1, MMP-7, PAI-1, FSP1, and HGF, which consequently leads to fibroblast activation, EMT, and renal fibrosis (Edeling et al., 2016; Zhou et al., 2017). In RCC, the activation of WNT signaling through WNT ligand secretion, WNT receptor overexpression, and function loss of WNT antagonists has been reported to promote tumorigenesis and metastasis (Xu et al., 2016). Ligands of the WNT canonical pathway, such as WNT1 and WNT10A, are associated with tumor progression, poor prognosis, and tumor invasiveness in ccRCC patients (Kruck et al., 2013; Piotrowska et al., 2020) by oncogene activation, such as c-Myc and cyclin D1 (Furge et al., 2007; Karim et al., 2016). On the contrary, some ligands of the WNT non-canonical pathway, including WNT5A and WNT7A, demonstrate a potential tumor-suppressive role in renal cancer (Tamimi et al., 2008; Kondratov et al., 2012). In addition, the functional loss of WNT antagonists is another vital trigger of WNT signaling activation in RCC. Early in 2006, Urakami et al. (2006) reported that RCC patients significantly demonstrated high methylation levels of WNT antagonists, including sFRP-1, sFRP-2, sFRP-4, sFRP-5, WIF-1, and Dkk-3, compared to healthy controls. Specifically, the methylation level of sFRP-1 serves as an independent biomarker for RCC prognosis. Later, the same team also identified that WIF-1, a WNT antagonist belonging to the secreted frizzled-related protein (sFRP) class, functions as a tumor suppressor in RCC cells. Overexpression of WIF-1 enhances RCC cell apoptosis and inhibits tumor growth *in vivo* (Kawakami et al., 2009). A similar antitumor potential has also been identified in IGFBP-4, DKK-1, and DKK-3 (Ueno et al., 2011; Xu et al., 2017; Chen et al., 2018).

**TABLE 1:** Fibrotic signaling in RCC.

Key molecule	Associated pathway	Mechanism	Biological function
CAF-mediated fibrotic signaling in RCC			
LOX/LOX2	ECM remodeling	Promotes collagen stiffness increment and integrin stabilization and fiber formation and suppresses the protease and proteasome system	RCC invasion and metastasis
MMP-2/9	AKT/NF-κB/MMP-9 and collagen I/MMP-2	Degrades ECM proteins; proteolytic breakdown of tissue barriers to invasion; and promotes circulating tumor cell extravasation	RCC invasion and metastasis
MMP-13	TGF-β1/MMP-13	Promotes osteoblastic matrix degradation and osteoclastic activation	Bone metastasis
SDF-1	SDF-1/CXCR4 interaction	Promotes angiogenesis and organ metabolism	RCC proliferation and invasion
Collagen I	Upregulating MMP-2, ZEB2, and SNAIL	Facilitates EMT	Enhances RCC proliferation, adhesion, and migration
Fibronectin	Fibronectin/TGF-β1/Src/cyclin D1 and vimentin	Interacts with integrin α5 and integrin β1; promotes RCC cell migration; and promotes cyclin D1 and vimentin expression, TGF-β1 production, and Src and Smad phosphorylation	Enhances RCC cell growth and migration
Lactate	Aerobic glycolysis	Energy supply to the tumor; TME acidification; suppresses T-cell anticancer activities; and accelerates angiogenesis	Enhances RCC aggressiveness
TGF-β-centered fibrotic signaling in RCC			
lncRNA ATB	TGF-β1/ATB/DNMT1/p53	Regulates DNMT1 to suppress p53 and promotes the expression of N-cadherin and vimentin	Enhances RCC cell proliferation and migration
lncRNA SPRY4-IT1	ND	ND	Promotes RCC cell proliferation, migration, and invasion
Other fibrotic signaling in RCC			
WNT1	WNT canonical pathway	Activating oncogenes c-Myc and cyclin D1	Promotes ccRCC progression and invasiveness
WNT10A			
WNT5A	WNT non-canonical pathway	Directly regulated by PAX2	Potentially related to blastemal predominant Wilms tumorigenesis
WNT7A		WNT7A hypermethylation due to genetic/epigenetic alterations and promotes ccRCC oncogenesis	ccRCC tumor suppressor
WIF-1	WNT antagonist	Loss function of WIF in ccRCC triggers WNT signaling activation and inhibits RCC apoptosis and proliferation	Enhances RCC cell apoptosis and inhibits tumor growth
mTOR	mTOR/MEK1/ERK and mTOR/MMK6/p38 MAPK	Induces pro-proliferation gene (cyclins and Myc) expression and suppresses the anti-proliferation p53/p16 axis	Promotes RCC tumorigenesis, proliferation, and metastasis
Notch2	Notch2/Jagged1 interaction	Notch2/Jagged1 interaction modifies histone and gene amplification of oncogenesis-related genes.	Promotes ECC proliferation and metastasis

Activation of the mTOR signaling cascade also plays a pro-fibrotic role during chronic kidney injury. In diabetic nephropathy, induced glomerular mesangial hypertrophy and matrix expansion were mediated by TGF-β1 and its downstream AKT/PRAS40/mTOR axis in glomerular mesangial cells (Dey et al., 2012; Maity et al., 2020). Further studies confirmed that the activation of either mTOR1 or mTOR2 promotes fibroblast activation and myofibroblast proliferation (Jiang et al., 2013; Li et al., 2015). Moreover, the activation of mTOR2 facilitates macrophage polarization toward the pro-fibrotic M2 phenotype through the Rictor/mTORC2/AKT axis in the UUO mouse model (Ren et al., 2017). Both activated myofibroblasts and M2 macrophages are the main contributors to collagen production and ECM deposition during renal fibrosis. Intriguingly, downstream cascades of mTOR signaling also contribute to RCC oncogenesis and metastasis. The activation of mTOR is correlated with poor prognosis and aggressive tumorigenesis in RCC (Pantuck et al., 2007; Damayanti et al., 2018). Wu et al. (2021b) further uncovered that the activation of the mTOR pathway initiated RCC development from renal proximal tubular cells. Mechanistically, activated mTOR signaling upregulates MEK1 expression and promotes ERK activation, which induces pro-proliferation cyclins and Myc expression. Meanwhile, activated mTOR also suppresses the anti-proliferation p53/p16 axis *via* MKK6/p38 MAPK signaling. As a result, the TSC1 or VHL mutation-induced pathologic mTOR activation and its downstream cascades lead to renal cyst formation and RCC carcinogenesis [Wu et al. (2021b)]. Other mTOR-regulated molecules, such as autophagy-related light chain (LC3), fatty acids, EMT-associated eIF4E-binding protein 1 (4E-BP1), and ribosomal S6 kinase (S6K), were also found to contribute to RCC development and invasion (Dey et al., 2019; Qu et al., 2020; Tao et al., 2020).

The pro-fibrotic Notch signaling pathway also contributes to tumorigenesis. The interaction of Notch2 with its receptor Jagged1 not only promotes renal fibrosis and metabolism by regulating TFAM and PGC1-α expression (Han et al., 2017; Huang et al., 2018) but is also associated with RCC proliferation and metastasis through histone modification and gene amplification of cell fate determination features, *KLF4* and *SOX9*, renal development-related features, *PAX2* and *SALL1*, the stem cell maintenance-associated features, *PROM1* and *ALDH1A*, and chromatin modification-related feature, *MYST3* (Fendler et al., 2020).

Efforts have also been made to identify how RCC-specific fibrotic signaling differs from that of other types of cancer. Yang et al. (2021) identified 11 cytokines that are significantly associated with fibrosis in ccRCC, including brevican, prolactin, presenilin 1, and GRO. The team further found that ccRCC expressed a distinctly higher level of prolactin and prolactin receptors, compared to other malignant tumors like lung, liver, and breast cancers. Such ligand–receptor interactions are also correlated with the prognosis of ccRCC patients.

## 5 Therapeutic implications targeting fibrosis signaling

Due to the highly fibrotic feature of the RCC intratumoral environment, therapies targeting fibrosis signaling are exploited to suppress the progression of RCC (Table 2). In 2009, the mTORC1 inhibitors everolimus and temsirolimus had already been approved by the U.S. Food and Drug Administration (FDA) as single agents in the second-line setting and in the first-line in RCC patients’ treatment at advanced stages (Hudes et al., 2007; Motzer et al., 2008). Interestingly, another mTORC1/2 dual inhibitor AZD-2014 was also able to downregulate HIF-1α/2α and cyclin D expression and further inhibit RCC cell proliferation preclinically (Zheng et al., 2015). However, the phase II randomized control study among 49 patients with VEGF-refractory metastatic ccRCC showed that AZD-2014 was less toxic but also less effective than everolimus in improving patients’ overall survival and preventing tumor progression (Powles et al., 2016). Roulin et al. (2011) revealed that combined treatment with sorafenib and NVP-BEZ235, a novel dual PI3K/mTOR inhibitor, demonstrated enhanced antitumor efficacy in RCC cell lines, 786-0 and Caki-1, compared to either of the single treatments. Other mTOR inhibitors, such as mTORC2 inhibitors, PP242 and PP30 (Feldman et al., 2009; Li et al., 2021), and the mTORC1/2 dual inhibitor, WYE-125132 (Yu et al., 2010), all demonstrated ideal antitumor capacity in RCC preclinically. The aforementioned findings provide new insights into the development of mTOR-targeted novel antitumor therapeutic strategies for RCC.

**TABLE 2:** Anticancer therapies targeting fibrosis in RCC.

Drug	Target	Mechanism	Type	Current status
Target mTOR signaling				
Everolimus	mTORC1	Selective inhibition of mTORC1	Small-molecule inhibitor	FDA approved
Temsirolimus	mTORC1	Selective inhibition of mTORC1	Small-molecule inhibitor	FDA approved
AZD2014	mTORC1/mTORC2	Dual inhibition of mTORC1 and mTORC2	Small-molecule ATP competitive inhibitor	Phase II trial completed
NVP-BEZ235 combined with sorafenib	PI3K/mTOR	Dual inhibition of PI3K/Akt/mTOR	Small-molecule ATP competitive inhibitor	Preclinical study conducted on RCC cell lines
PP242 and PP30	mTORC2	Selective inhibition of mTORC2	Small-molecule ATP competitive inhibitor	Preclinical study conducted on RCC cell lines (UMRC6, 786-0, and UOK121)
WYE-125132	mTORC1/mTORC2	Dual inhibition of mTORC1 and mTORC2	Small-molecule ATP competitive inhibitor	Preclinical study conducted on the mouse RCC model
Target TGF-β signaling				
Fresolimumab	TGF-β	Human TGF-β1/β2/β3 neutralizer	Human monoclonal antibody	Phase I trial completed
LY3022859	TGF-β2	Human TGF-β2 neutralizer	Human monoclonal antibody	Phase I trial completed
Valproic acid	Smad4	Smad4 suppressor		Preclinical study conducted on RCC cell lines (786-0 and Caki-1)
Pirfenidone	TGF-β	TGF-β inhibitor	Broad-based anti-fibrotic drug	Preclinical study conducted on the mouse RCC model

The classic pro-fibrosis TGF-β signaling pathway is another promising therapeutic target of RCC. Early in 2014, a phase I clinical study used the human TGF-β1/β2/β3 neutralizer GC1008 (fresolimumab) to treat patients with advanced malignant melanoma and RCC (Morris et al., 2014). However, only seven out of the total 29 patients achieved a partial response. Later, in 2017, another phase I study among advanced cancer patients using the TGF-β receptor 2 monoclonal antibody LY3022859 failed to determine the maximum tolerated dose due to adverse side effects (Tolcher et al., 2017). The difficulties in the clinical application of anti-TGF-β antibodies among cancer patients might be related to the dual functions of the diverse TGF-β-dependent downstream cascades (Meng et al., 2012a). Smad3, one of the key downstream transcription factors of TGF-β signaling, plays a major pathogenic role during renal fibrosis and inflammation. However, the activation of TGF-β-dependent Smad2 and Smad7 pathways demonstrates anti-fibrosis and renal protective effects (Meng et al., 2012b). Nevertheless, efforts have been continuously made to improve the therapeutic effects of TGF-β-targeted treatments. Strauss et al. (2018) led another phase I study using a novel bifunctional fusion protein M7824 against PD-L1 and TGF-β among patients with an advanced solid tumor, and this novel targeted agent demonstrated encouraging antitumor effects with a good safety profile (Strauss et al., 2018). Previously, Lian et al. (2018) used a Smad7 inducer asiatic acid (AA) in combination with a Smad3 inhibitor naringenin (NG) to restore the balance of Smad3 and Smad7 signaling pathways in invasive melanoma and lung cancer mouse models and achieved significant anticancer effects by enhancing natural killer (NK) cell-mediated cytotoxicity through attenuating Smad3-induced suppression on two transcription factors essential for NK development and functions, namely, ID2 and IRF2. In addition, the same group discovered SIS3, a small-molecule inhibitor of Smad3, which effectively delays tumor development by suppressing TGF-β-mediated angiogenesis and immune escape in lung cancer (Tang et al., 2017; Lian et al., 2022). Although there is still very limited clinical evidence, preclinical studies have already revealed the promising anti-RCC potential of TGF-β signal-targeted therapies. A study using valproic acid, a Smad4 suppressor, significantly decreased cancer cell viability by inducing cell apoptosis and inhibiting EMT marker (E-cadherin and vimentin) expression in RCC cell lines (Mao et al., 2017). More recently, the anti-fibrotic drug pirfenidone (PFD), which has been approved by the FDA for the treatment of renal fibrosis, has been shown to be capable of suppressing RCC progression *in vivo* (Wang et al., 2022a). Mechanistically, PFD significantly downregulates TGF-β production in the RCC mouse model, thus mitigating TGF-β-mediated EMT and immunosuppressive MDSC infiltration into the TME.

## 6 Conclusion

Fibrosis has long been recognized as a major contributor to cancer progression and invasion. In this review, we systematically summarized the clinical association between renal fibrosis and poorer RCC outcomes and how a fibrotic microenvironment interacts with RCC in the form of ITF, CAFs, and PC fibrosis. In addition, we further conducted transcriptomic analysis on an up-to-date scRNA-seq profile of human RCC to confirm the participation of diverse fibrotic signaling in RCC development at single-cell resolution. The crosstalk between common pro-fibrotic pathways, including TGF-β, WNT, mTOR, and NOTCH signaling pathways, and RCC has also been discussed. In conclusion, the discovery of the mechanisms through which fibrotic signaling promotes tumorigenesis and aggressiveness in RCC provides inspiration for the development of anti-fibrotic therapies, such as mTOR inhibitors or anti-TGF-β antibodies, as novel therapeutic strategies for renal cancer.
